# Synthesis, crystal structure, Hirshfeld surface and void analysis of bis­(μ_2_-4-amino­benzoato-κ^2^ *O*:*O*′)bis­[bis­(4-amino­benzoato-κ^2^ *O*,*O*′)di­aquathulium(III)] dihydrate

**DOI:** 10.1107/S2056989022001116

**Published:** 2022-02-03

**Authors:** Kasumova Samira Ali, Muhammad Ashfaq, Muhammad Nawaz Tahir, Elman Muhammad Movsumov, Khurram Shahzad Munawar

**Affiliations:** a Azerbaijan State Aqrarian University, Ganja City, Azerbaijan; b Department of Physics, University of Sargodha, Sargodha, Pakistan; c Department of Physics, University of Minawali, Miamwali, Pakistan; d Department of Chemistry, University of Sargodha, Sargodha, Pakistan; e Department of Chemistry, University of Minawali, Miamwali, Pakistan

**Keywords:** *p*-amino­benzoic acid, dinuclear thulium complex, single crystal, Hirshfeld analysis, crystal structure

## Abstract

The asymmetric unit of the title compound, [Tm_2_(C_7_H_6_NO_2_)_6_(H_2_O)_4_]·2H_2_O, contains three 4-amino­benzoate (**4ABA**) ligands, two coordinated water mol­ecules, a thulium metal ion, and a water mol­ecule of crystallization. The overall structure of the complex (**4ABA-Tm**) is in the form of a dimer. In the dinuclear thulium complex, symmetry-relevant TmO_8_ coordination polyhedra are formed by the O atoms of two chelating 4-amino­benzoate ligands, the O atoms of two non-chelating 4-amino­benzoate ligands, and two water mol­ecules. The Tm—O bond lengths range from 2.216 (3) to 2.471 (3) Å with the Tm⋯Tm separation in the dinuclear complex being 4.7863 (5) Å. The crystal structure features O—H⋯N, N—H⋯O, and O—H⋯O hydrogen-bonding inter­actions. Further stabilization of the crystal packing is due to C—H⋯π and off-set π–π stacking inter­actions. Hirshfeld surface analysis indicates that H⋯H contacts are the most significant contributors to the crystal packing (45.9%). In addition, a void analysis was performed to check the strength of the crystal packing.

## Abstract 2

The asymmetric unit of the title compound comprises three 4-amino­benzoate ligands, two coordinated water mol­ecules, a thulium metal ion and a water mol­ecule of crystallization. The crystal structure features O—H⋯N, N—H⋯O, and O—H⋯O hydrogen-bonding inter­actions as well as C—H⋯π and off-set π–π stacking inter­actions. Hirshfeld surface analysis indicates that H⋯H contacts are the most significant contributors to the crystal packing.

## Chemical context

The coordination chemistry of rare-earth metals has been widely studied, and the structures of a significant variety of complexes with diverse kinds of ligands have been reported (You *et al.*, 2021 ▸). In particular, the lanthanide contraction along the series is of inter­est, and in a detailed analysis of this phenomenon using elements from the lanthanide series, *p*-amino­benzoic acid (H*L*) was found to be a very useful and biologically important ligand (Smith & Lynch, 2015 ▸). The carboxyl­ate group of H*L* can be coordinated with the metals simultaneously in three different modes, namely chelating, bridging, and chelating-bridging (Ali *et al.*, 2014 ▸). In the complexes of H*L* with alkali metals such as Na^+^ or K^+^, the ligand is not directly coordinated to the metal ion, but rather it is surrounded by coordinated water mol­ecules (You *et al.*, 2021 ▸). Both the carb­oxy­lic and amino groups of the ligand are coordinated to the metal in complexes with Ba^2+^, Ag^+^, Zn^2+^, Cd^2+^, and Ni^2+^ (Mamedov *et al.*, 1982 ▸; Amirasłanov *et al.*, 1982*a* ▸), while only the oxygen atoms of the carb­oxy­lic groups are coordinated to the metal ion in complexes of Sr^2+^, Mg^2+^, and Co^2+^ with this ligand (Amirasłanov *et al.*, 1982*b* ▸; Sun *et al.*, 2004 ▸). In comparison to the above coordination diversity, in the complexes of H*L* with rare-earth elements like Nd^+3^ and Sm^+3^ (Khiyalov *et al.*, 1981 ▸; Mao & Lianq, 2016 ▸), only the nitro­gen atom of the amino group is coordinated by the central metal atom, while in complexes of Lu^+3^ and Ho^+3^ with H*L* (Sun *et al.*, 2004 ▸), the nitro­gen atom of the amino group is not coordinated while the ligands are attached to the metal atom by the oxygen atoms of the carboxyl­ate moiety. In this context, we report the synthesis, crystal structure, Hirshfeld surface, void, thermogravimetric and FT–IR analysis of the title compound, [Tm_2_(C_7_H_6_NO_2_)_6_(H_2_O)_4_]·2H_2_O, which is closely related to its Lu^+3^ and Ho^+3^ analogues (Sun *et al.*, 2004 ▸). 
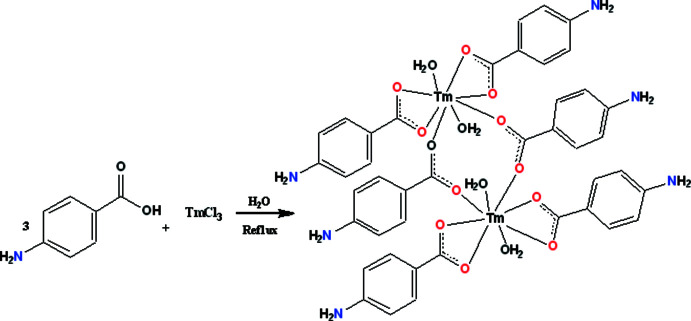


## Structural commentary

**Figure 1:**
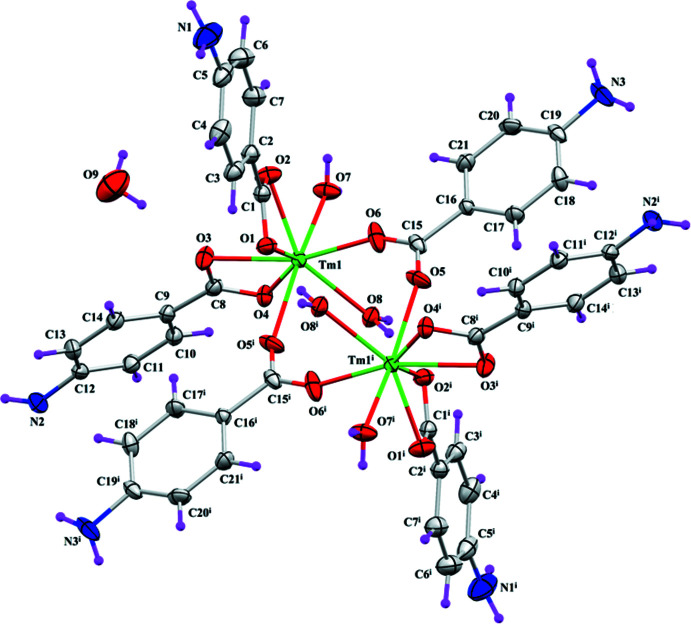
*ORTEP* view of **4ABA-Tm** with ellipsoids drawn at a 30% probability level with H atoms shown as small circles of arbitrary radii.

The asymmetric unit of the title compound **4ABA-Tm** (Fig. 1 ▸) contains a centrosymmetric thulium dinuclear complex and one water mol­ecule of crystallization. Each Tm^III^ atom is octa­coordinated by two chelating 4-amino­benzoate ligands, two bridging 4-amino­benzoate ligands and two coordinated water mol­ecules. In the coordination sphere, bond lengths range from 2.216 (3) to 2.471 (3) Å, while bond angles range from 53.82 (10) to 161.28 (12)° (Table 1 ▸). The Tm⋯Tm^i^ separation in the **4ABA-Tm** complex is 4.7863 (5) Å (Table 1 ▸). The oxygen atoms O2 of the first 4-amino­benzoate chelate (ligand *A*, C1–C7/N1/O1/O2), O4 of the second 4-amino­benzoate chelate (ligand *B*, C8–C14/N2/O3/O4) and O5 of the 4-amino­benzoate non-chelate (ligand *C*, C15–C21/N3/O5/O6) show maximum deviations from their respective planes with values of 0.1748 (3) Å for O2, 0.3087 (3) Å for O4, and 0.1351 (3) Å for O5. Ligand *B* is twisted at a dihedral angle of 70.83 (7)° with respect to ligand *A*. The non-chelating ligand *C* is twisted at dihedral angles of 79.7 (9) and 72.7 (9)°, respectively, to the planes of ligands *A* and *B*. Intra­molecular O—H⋯O hydrogen bonding (Table 2 ▸) involving OH from the non-coordinating water and the O atom (hydrogen-bond acceptor) of the chelating 4-amino­benzoate ligand stabilizes the mol­ecular configuration.

## Supra­molecular features

**Figure 2:**
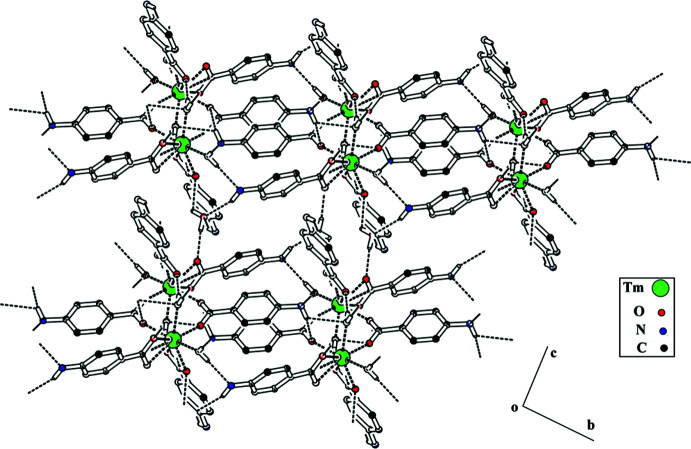
Packing diagram of **4ABA-Tm**. Selected H atoms are shown for clarity.

**Figure 3:**
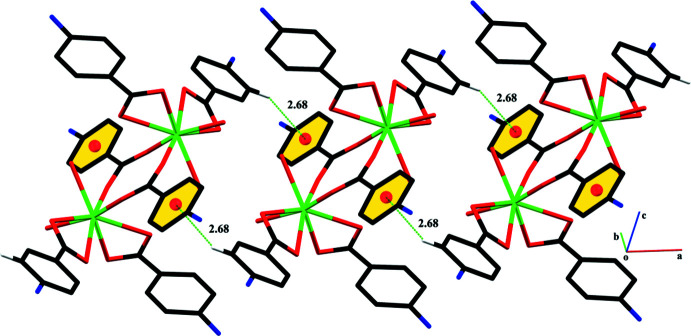
Graphical representation of C—H⋯π inter­actions in **4ABA-Tm**. Selected H atoms are shown while the water mol­ecules are omitted for clarity.

**Figure 4:**
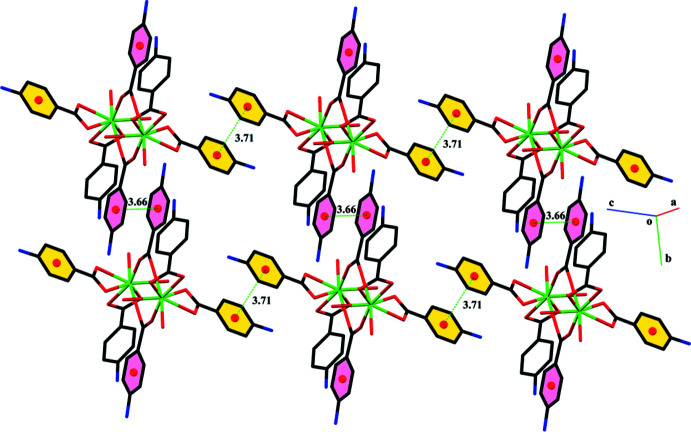
Graphical representation of off-set π–π inter­actions in **4ABA-Tm**. H atoms and water mol­ecules are not shown for simplicity.

The centrosymmetric dinuclear thulium complexes are linked through O—H⋯O, O—H⋯N and N—H⋯O hydrogen-bonding inter­actions. A *C*11 chain running along the *b*-axis direction is formed through O7—H7*A*⋯N3 H bonds while a loop is formed through the O7—H7*A*⋯N3 inter­actions. The water mol­ecule of crystallization plays an important role in the stabilization of the crystal packing, acting as a hydrogen-bond donor and as well as a hydrogen-bond acceptor, connecting the centrosymmetric dinuclear thulium complex with each other. The hydrogen bonds lead to the formation of layers parallel to the *bc* plane (Fig. 2 ▸, Table 2 ▸). These layers are linked through C—H⋯π (Fig. 3 ▸), with H⋯π distance of 2.68 Å and off-set π–π stacking inter­actions (Fig. 4 ▸) with inter-centroid distances ranging from 3.661 (3) to 3.709 (3) Å, forming a three-dimensional network.

## Hirshfeld surface analysis

**Figure 5:**
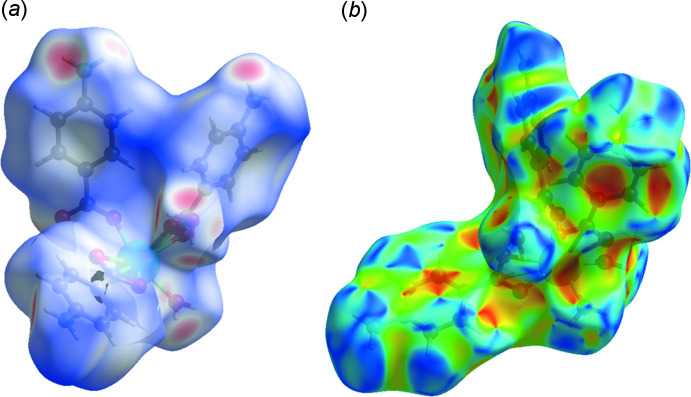
HS plotted over (*a*) *d*_norm_ in the range −1.073 to 1.740 a.u. and (*b*) shape-index in the range −1 to 1 a.u.

A Hirshfeld surface (HS) analysis was carried out using *Crystal Explorer 21.5* (Spackman *et al.*, 2021 ▸) in order to explore the non-covalent inter­actions in terms of the Hirshfeld surface and two-dimensional fingerprint plots. The HS of a mol­ecule is the region in the crystal where the electron density relevant to the promolecule is greater than the electron density relevant to the procrystal (Spackman *et al.*, 2009 ▸; Ashfaq *et al.*, 2020 ▸). The Hirshfeld surface is constructed by employing colour coding to show the inter­atomic contacts that are shorter (red areas), equal to (white areas), or longer than (blue areas) the sum of the van der Waals radii (Ashfaq *et al.*, 2021*a* ▸,*b* ▸). The red spots on the surface mapped over *d*_norm_ (Fig. 5 ▸ *a*) indicate the involvement of atoms in hydrogen-bonding inter­actions. The HS mapped over shape-index (Fig. 5 ▸ *b*) is used to check for the presence of inter­actions such as C—H⋯π and π–π stacking (Ashfaq *et al.*, 2021*a* ▸,*b* ▸). The existence of adjacent red and blue triangular regions around the aromatic rings conforms to the presence of π–π stacking inter­actions in the title compound.

**Figure 6:**
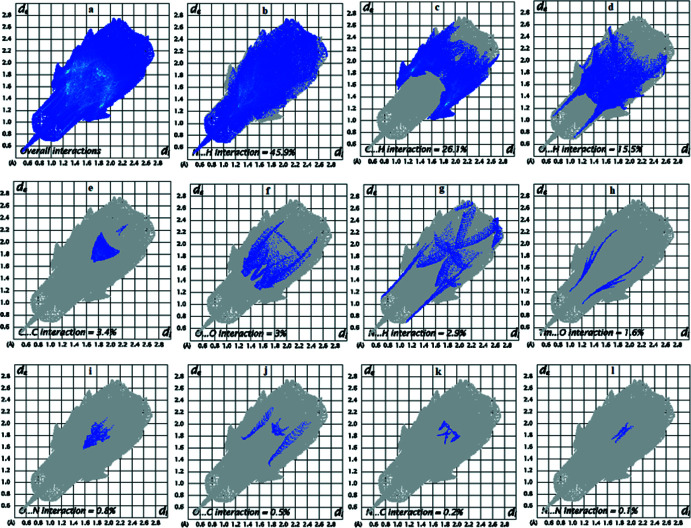
Two-dimensional fingerprint plots of **4ABA-Tm** for (*a*) all inter­actions and (*b*)–(*l*) individual inter­atomic contacts.

Two-dimensional fingerprint plots provide unique information about the non-covalent inter­actions and the crystal packing in terms of the percentage contribution of the inter­atomic contacts (Spackman *et al.*, 2002 ▸; Ashfaq *et al.*, 2021*a* ▸,*b* ▸ ▸). Fig. 6 ▸ *a* shows the two-dimensional fingerprint plot for the overall inter­actions in **4ABA-Tm** where *d*_i_ and *d*_e_ are the distances from the Hirshfeld surface to the nearest atom inside the Hirshfeld surface and outside it, respectively. The most important inter­atomic contact is H⋯H (Fig. 6 ▸ *b*) as it makes the highest contribution to the crystal packing (45.9%). Other major contributors are C⋯H (26.1%, Fig. 6 ▸ *c*) and O⋯H (15.5%, Fig. 6 ▸ *d*) inter­actions. The inter­atomic contacts that make comparatively smaller contributions in the crystal packing are shown in Fig. 6 ▸ *e*–*l*.

**Figure 7:**
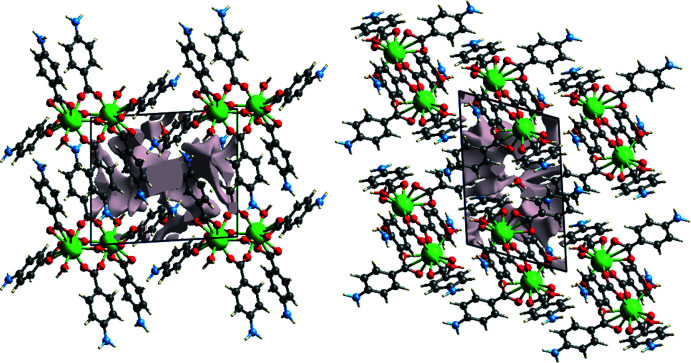
Graphical representation of **4ABA-Tm in** (*a*) a view along the *a* axis and (*b*) a view along the *b* axis.

The response to applied stress or force mainly depends on the strength of the crystal packing in single crystals, which have a high mechanical strength as the mol­ecules are strongly packed into them. To check whether the title compound is mechanically stable or not, a void analysis was performed. In order to calculate voids in the crystal packing, the electron densities of all of the atoms in the mol­ecules present in the asymmetric unit are added up, the atoms being assumed to be spherically symmetric (Turner *et al.*, 2011 ▸; Kargar *et al.*, 2022 ▸). The volume of the void in the crystal packing of the title compound is 120.81 Å^3^ (Fig. 7 ▸), which infers that voids occupy 10.51% of the space and, hence, the mol­ecules are strongly packed in the title compound.

## Infra-red spectroscopy

The structure of the newly synthesized complex was also investigated by FT–IR spectroscopy. It was found that the absorption bands of the –NH_2_ group appeared in the region of 3200 cm^−1^ while the absorption bands due to Tm—OH_2_ are visible in the region of 325 cm^−1^. The aromatic carbons show their absorption band at 1225 cm^−1^, while the Tm—O band is visible in the region of 650 cm^−1^. The absorption bands observed in the FT–IR spectrum of the free ligand in the regions of 1715 and 1435 cm^−1^ are caused by symmetric (ν_s_) and asymmetric (ν_as_) stretching of the carboxyl group, which are shifted to 1635 and 1436 cm^−1^, respectively, upon coordination with the Tm^III^ metal ion. The difference between ν_s_ and ν_as_ is 199 cm^−1^, indicating that the carboxyl groups are coordinated to the central metal ion by chelate and bidentate-bridging coordination modes.

## Thermogravimetric analysis

The title complex was further characterized by thermogravimetry. Thermolysis occurs in three stages. In the first stage, at a temperature of 20–200°C, inter­molecular and coordinated water mol­ecules are released, with a weight loss of 4.69%. The complex remains stable over the temperature 200–400°C. In the second stage, at a temperature of 400–600°C, the hydro­carbon residues are decomposed and simultaneously burned out. Thulium carbonate is formed in the last stage at a temperature between 600 and 800°C. The final product of decomposition above 800°C is metal oxide.

It is known that lanthanide carboxyl­ates have good spectroscopic characteristics; they have enhanced thermal stability and are also resistant to moisture and oxygen in the air, which is of great importance in the production and operation of photoluminescent and electroluminescent devices based on them.

## Database survey

A search of the Cambridge Structural Database (CSD, version 5.40; update February 2021; Groom *et al.*, 2016) gave 206 hits, some of whose crystal structures are closely related to **4ABA-Tm**. These include the yttrium (NADYEX), holmium (NADZAU), lutetium (NADZIC) and ytterbium (YENRAK01) complexes reported by Sun *et al.* (2004 ▸). Erbium (YUTNAE; Smith & Lynch, 2015 ▸) and terbium (NADXEW01; Ye *et al.*, 2004 ▸) complexes were also found in the literature.

## Synthesis and crystallization

The infra-red spectrum of **4ABA-Tm** in the range 4000 to 250 cm^−1^ was recorded on an FT–IR Prestige 21 spectrophotometer after preparing the samples with KBr pellets. Thermal analysis was carried out using a NETSCHSTA-409 PC/PG derivatograph, TG, DTG and DTA curves were obtained in a static air atmosphere at a heating rate of 10°C min^−1^ from 20–800°C using platinum crucibles. Highly sintered Al_2_O_3_ was used as a reference. The elemental analysis for C, H, and N was performed using a Costech ECS 4010 CHNSO analyzer.

**Preparation of the title complex**

The reaction of aqueous solutions of TmCl_3_ and sodium *p*-amino­benzoate (1:3) yielded single crystals of tris-(*p*-amino­benzoato)thulium(III) dihydrate suitable for X-ray diffraction analysis. The mixture was refluxed for 30 minutes and then cooled to room temperature. After filtration, the filtrate was left for several days, covered with aluminum foil, until yellow prismatic crystals appeared. C_42_H_48_N_6_O_18_Tm_2_, *M*: 1262.72 g mol^−1^. Elemental analysis: calculated %: C:41.11; N: 6.25; Tm: 27.57: found %: C:41.24; N:6.72; Tm: 27.41.

## Refinement

Crystal data, data collection and structure refinement details are summarized in Table 3 ▸. H atoms of all the water mol­ecules and the amino groups of 4-amino­benzoate ligands were found by the careful inspection of residual electron-density peaks and positional parameters were refined using bond-length restraints (O—H = 0.82 Å, N—H = 0.85 Å) with *U*_iso_(H) = 1.5U_eq_(O) or 1.2*U*_eq_(N). All other H atoms were refined at calculated positions using a riding-model approximation [C—H = 0.93 Å, *U*_iso_(H) = 1.2*U*_eq_(C)]. The highest positive and negative features in the final difference map are within 0.83 Å of the Tm atom.

## Supplementary Material

Crystal structure: contains datablock(s) global, I. DOI: 10.1107/S2056989022001116/zn2014sup1.cif

Structure factors: contains datablock(s) I. DOI: 10.1107/S2056989022001116/zn2014Isup3.hkl

CCDC reference: 2118339

Additional supporting information:  crystallographic information; 3D view; checkCIF report

## Figures and Tables

**Table 1:** Selected geometric parameters (Å, °)

Tm1—O5	2.216 (3)	Tm1—O3	2.374 (3)
Tm1—O6^i^	2.223 (3)	Tm1—O8	2.382 (3)
Tm1—O7	2.293 (3)	Tm1—O4	2.457 (3)
Tm1—O2	2.329 (3)	Tm1—O1	2.471 (3)
			
O5—Tm1—O6^i^	108.53 (13)	O3—Tm1—O8	124.48 (11)
O5—Tm1—O7	156.90 (13)	O5—Tm1—O4	80.34 (11)
O6^i^—Tm1—O7	84.40 (13)	O6^i^—Tm1—O4	142.65 (11)
O5—Tm1—O2	125.29 (12)	O7—Tm1—O4	78.06 (11)
O6^i^—Tm1—O2	81.97 (13)	O2—Tm1—O4	123.35 (10)
O7—Tm1—O2	74.42 (11)	O3—Tm1—O4	53.82 (10)
O5—Tm1—O3	80.05 (12)	O8—Tm1—O4	72.41 (10)
O6^i^—Tm1—O3	161.28 (12)	O5—Tm1—O1	75.80 (11)
O7—Tm1—O3	93.61 (13)	O6^i^—Tm1—O1	76.29 (11)
O2—Tm1—O3	79.58 (11)	O7—Tm1—O1	126.75 (11)
O5—Tm1—O8	78.30 (11)	O2—Tm1—O1	54.20 (10)
O6^i^—Tm1—O8	74.11 (12)	O3—Tm1—O1	90.29 (10)
O7—Tm1—O8	87.42 (11)	O8—Tm1—O1	131.47 (10)
O2—Tm1—O8	151.26 (11)	O4—Tm1—O1	139.92 (9)

Symmetry code: (i) 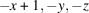 .

**Table 2:** Hydrogen-bond geometry (Å, °) *Cg*3 is the centroid of the C16–C21 ring.

*D*—H⋯*A*	*D*—H	H⋯*A*	*D*⋯*A*	*D*—H⋯*A*
O7—H7*A*⋯N3^ii^	0.82 (1)	1.95 (2)	2.753 (6)	169 (5)
O7—H7*B*⋯N2^iii^	0.82 (1)	2.18 (2)	2.940 (5)	154 (5)
O8—H8*A*⋯O1^i^	0.82 (1)	1.98 (1)	2.791 (4)	172 (4)
O8—H8*B*⋯O4^iv^	0.82 (1)	1.96 (1)	2.780 (4)	174 (4)
N1—H1*B*⋯O9^v^	0.85 (1)	2.06 (3)	2.870 (8)	158 (7)
N2—H2*B*⋯O9^vi^	0.85 (1)	2.24 (2)	3.051 (7)	161 (5)
N3—H3*B*⋯O5^vii^	0.84 (1)	2.46 (3)	3.173 (6)	144 (5)
N3—H3*B*⋯O8^vii^	0.84 (1)	2.56 (5)	3.092 (5)	122 (4)
O9—H9*A*⋯O3	0.83 (1)	2.00 (1)	2.828 (6)	172 (7)
O9—H9*B*⋯O2^viii^	0.84 (1)	2.34 (6)	2.849 (6)	119 (5)
C11—H11⋯*Cg*3^ix^	0.93	2.68	3.538 (5)	155

Symmetry codes: (i) 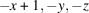 ; (ii) 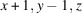 ; (iii) 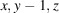 ; (iv) 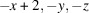 ; (v) 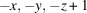 ; (vi) 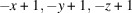 ; (vii) 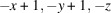 ; (viii) 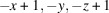 ; (ix) 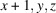 .

**Table 3:** Experimental details

Crystal data	
Chemical formula	C_42_H_48_N_6_O_18_Tm_2_
*M*_r_	1262.72
Crystal system, space group	Triclinic, *P* 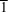
Temperature (K)	296
*a*, *b*, *c* (Å)	8.9659 (6), 10.9722 (7), 12.8027 (8)
α, β, γ (°)	88.195 (3), 71.599 (3), 74.402 (3)
*V* (Å^3^)	1149.10 (13)
*Z*	1
Radiation type	Mo *K*α
μ (mm^−1^)	3.92
Crystal size (mm)	0.32 × 0.18 × 0.16

Data collection	
Diffractometer	Bruker Kappa APEXII CCD
Absorption correction	Multi-scan (*SADABS*; Krause et al., 2015 ▸)
*T*_min_, *T*_max_	0.983, 0.986
No. of measured, independent and observed [*I* > 2σ(*I*)] reflections	13239, 4891, 4358
*R*_int_	0.037
(sin θ/λ)_max_ (Å^−1^)	0.639

Refinement	
*R*[*F* ^2^ > 2σ(*F* ^2^)], *wR*(*F* ^2^), *S*	0.029, 0.064, 1.04
No. of reflections	4891
No. of parameters	343
No. of restraints	19
H-atom treatment	H atoms treated by a mixture of independent and constrained refinement
Δρ_max_, Δρ_min_ (e Å^−3^)	1.80, −1.07

Computer programs: *APEX2* and *SAINT* (Bruker, 2007 ▸), *SHELXS97* (Sheldrick, 2008 ▸), *SHELXL2018/3* (Sheldrick, 2015 ▸), *ORTEP-3 for Windows* *WinGX* (Farrugia, 2012 ▸) and *PLATON* (Spek, 2020 ▸).

## supplementary crystallographic information

### Crystal data

**Table:**

C_42_H_48_N_6_O_18_Tm_2_	*Z* = 1
*M**_r_* = 1262.72	*F*(000) = 624
Triclinic, *P*1	*D*_x_ = 1.825 Mg m^−^^3^
*a* = 8.9659 (6) Å	Mo *K*α radiation, λ = 0.71073 Å
*b* = 10.9722 (7) Å	Cell parameters from 4358 reflections
*c* = 12.8027 (8) Å	θ = 2.5–27.0°
α = 88.195 (3)°	µ = 3.92 mm^−^^1^
β = 71.599 (3)°	*T* = 296 K
γ = 74.402 (3)°	Prism, light yellow
*V* = 1149.10 (13) Å^3^	0.32 × 0.18 × 0.16 mm

### Data collection

**Table:**

Bruker Kappa APEXII CCD diffractometer	4891 independent reflections
Radiation source: fine-focus sealed tube	4358 reflections with *I* > 2σ(*I*)
Graphite monochromator	*R*_int_ = 0.037
Detector resolution: 7.828 pixels mm^-1^	θ_max_ = 27.0°, θ_min_ = 2.5°
ω scans	*h* = −11→9
Absorption correction: multi-scan (SADABS; Krause et al., 2015)	*k* = −11→14
*T*_min_ = 0.983, *T*_max_ = 0.986	*l* = −16→16
13239 measured reflections	

### Refinement

**Table:**

Refinement on *F*^2^	Primary atom site location: structure-invariant direct methods
Least-squares matrix: full	Secondary atom site location: difference Fourier map
*R*[*F*^2^ > 2σ(*F*^2^)] = 0.029	Hydrogen site location: inferred from neighbouring sites
*wR*(*F*^2^) = 0.064	H atoms treated by a mixture of independent and constrained refinement
*S* = 1.04	*w* = 1/[σ^2^(*F*_o_^2^) + (0.0257*P*)^2^ + 0.6134*P*] where *P* = (*F*_o_^2^ + 2*F*_c_^2^)/3
4891 reflections	(Δ/σ)_max_ = 0.001
343 parameters	Δρ_max_ = 1.80 e Å^−^^3^
19 restraints	Δρ_min_ = −1.07 e Å^−^^3^

### Special details

**Table:**

Geometry. All esds (except the esd in the dihedral angle between two l.s. planes) are estimated using the full covariance matrix. The cell esds are taken into account individually in the estimation of esds in distances, angles and torsion angles; correlations between esds in cell parameters are only used when they are defined by crystal symmetry. An approximate (isotropic) treatment of cell esds is used for estimating esds involving l.s. planes.
Refinement. Refinement of *F*^2^ against ALL reflections. The weighted *R*-factor *wR* and goodness of fit *S* are based on *F*^2^, conventional *R*-factors *R* are based on *F*, with *F* set to zero for negative *F*^2^. The threshold expression of *F*^2^ > σ(*F*^2^) is used only for calculating *R*-factors(gt) *etc*. and is not relevant to the choice of reflections for refinement. *R*-factors based on *F*^2^ are statistically about twice as large as those based on *F*, and *R*- factors based on ALL data will be even larger.

### Fractional atomic coordinates and isotropic or equivalent isotropic displacement parameters (Å^2^)

**Table:**

	*x*	*y*	*z*	*U*_iso_*/*U*_eq_	
Tm1	0.63601 (2)	−0.01205 (2)	0.13511 (2)	0.02731 (7)	
O1	0.3377 (4)	0.0164 (3)	0.2017 (2)	0.0380 (7)	
O2	0.4938 (4)	−0.1008 (3)	0.2892 (2)	0.0458 (8)	
O3	0.6097 (4)	0.1341 (3)	0.2770 (2)	0.0468 (8)	
O4	0.8365 (3)	0.1015 (2)	0.1382 (2)	0.0351 (6)	
O5	0.5346 (4)	0.1682 (3)	0.0675 (3)	0.0518 (9)	
O6	0.3957 (5)	0.1706 (3)	−0.0479 (3)	0.0601 (10)	
O7	0.8305 (4)	−0.1673 (3)	0.1803 (3)	0.0469 (8)	
H7A	0.9297 (15)	−0.183 (4)	0.164 (4)	0.056*	
H7B	0.801 (5)	−0.221 (3)	0.221 (3)	0.056*	
O8	0.8252 (4)	−0.0313 (3)	−0.0465 (2)	0.0372 (7)	
H8A	0.783 (5)	−0.034 (4)	−0.094 (3)	0.045*	
H8B	0.9240 (14)	−0.053 (4)	−0.077 (3)	0.045*	
N1	−0.1604 (7)	−0.2330 (6)	0.5608 (4)	0.0795 (16)	
H1A	−0.163 (7)	−0.258 (6)	0.625 (2)	0.095*	
H1B	−0.248 (4)	−0.193 (6)	0.549 (5)	0.095*	
N2	0.8267 (6)	0.6323 (4)	0.3384 (3)	0.0520 (11)	
H2A	0.922 (3)	0.629 (5)	0.340 (4)	0.062*	
H2B	0.753 (4)	0.677 (4)	0.393 (3)	0.062*	
N3	0.1667 (6)	0.7572 (3)	0.1054 (4)	0.0595 (12)	
H3A	0.184 (7)	0.781 (5)	0.162 (3)	0.071*	
H3B	0.209 (6)	0.792 (5)	0.049 (2)	0.071*	
C1	0.3548 (6)	−0.0615 (4)	0.2755 (3)	0.0342 (9)	
C2	0.2186 (6)	−0.1081 (4)	0.3440 (3)	0.0350 (9)	
C3	0.0591 (6)	−0.0550 (4)	0.3473 (3)	0.0407 (10)	
H3	0.0364	0.0097	0.3013	0.049*	
C4	−0.0683 (6)	−0.0961 (5)	0.4179 (4)	0.0502 (12)	
H4	−0.1752	−0.0594	0.4191	0.060*	
C5	−0.0343 (7)	−0.1938 (5)	0.4876 (4)	0.0576 (15)	
C6	0.1255 (7)	−0.2498 (5)	0.4815 (4)	0.0558 (13)	
H6	0.1497	−0.3172	0.5248	0.067*	
C7	0.2475 (6)	−0.2070 (4)	0.4126 (4)	0.0474 (11)	
H7	0.3544	−0.2449	0.4110	0.057*	
C8	0.7365 (5)	0.1669 (4)	0.2249 (3)	0.0330 (9)	
C9	0.7643 (5)	0.2844 (4)	0.2605 (3)	0.0315 (9)	
C10	0.9155 (5)	0.3092 (4)	0.2208 (3)	0.0349 (9)	
H10	1.0038	0.2487	0.1744	0.042*	
C11	0.9373 (6)	0.4220 (4)	0.2489 (3)	0.0370 (10)	
H11	1.0401	0.4360	0.2225	0.044*	
C12	0.8074 (6)	0.5145 (4)	0.3160 (3)	0.0379 (10)	
C13	0.6544 (6)	0.4907 (4)	0.3559 (3)	0.0421 (11)	
H13	0.5658	0.5524	0.4006	0.051*	
C14	0.6336 (6)	0.3768 (4)	0.3299 (3)	0.0379 (10)	
H14	0.5318	0.3614	0.3588	0.046*	
C15	0.4332 (5)	0.2238 (3)	0.0213 (4)	0.0347 (9)	
C16	0.3553 (5)	0.3619 (3)	0.0476 (3)	0.0260 (8)	
C17	0.3834 (5)	0.4241 (4)	0.1289 (3)	0.0359 (10)	
H17	0.4461	0.3781	0.1700	0.043*	
C18	0.3196 (6)	0.5536 (4)	0.1497 (4)	0.0425 (11)	
H18	0.3377	0.5938	0.2056	0.051*	
C19	0.2295 (5)	0.6232 (3)	0.0884 (4)	0.0377 (10)	
C20	0.1950 (6)	0.5613 (4)	0.0106 (4)	0.0431 (11)	
H20	0.1283	0.6070	−0.0280	0.052*	
C21	0.2592 (5)	0.4313 (4)	−0.0104 (3)	0.0372 (10)	
H21	0.2370	0.3908	−0.0642	0.045*	
O9	0.4466 (6)	0.1607 (6)	0.5070 (4)	0.0969 (15)	
H9A	0.499 (8)	0.146 (6)	0.4401 (15)	0.116*	
H9B	0.491 (9)	0.094 (4)	0.532 (5)	0.116*	

### Atomic displacement parameters (Å^2^)

**Table:**

	*U*^11^	*U*^22^	*U*^33^	*U*^12^	*U*^13^	*U*^23^
Tm1	0.03208 (11)	0.02149 (9)	0.02871 (10)	−0.00610 (7)	−0.01104 (7)	−0.00037 (6)
O1	0.0419 (18)	0.0381 (15)	0.0329 (15)	−0.0097 (13)	−0.0122 (13)	0.0087 (12)
O2	0.0425 (19)	0.0590 (19)	0.0446 (18)	−0.0195 (16)	−0.0225 (15)	0.0214 (15)
O3	0.054 (2)	0.0528 (18)	0.0336 (17)	−0.0295 (16)	−0.0005 (15)	−0.0096 (14)
O4	0.0367 (17)	0.0360 (14)	0.0327 (15)	−0.0106 (13)	−0.0096 (13)	−0.0083 (12)
O5	0.049 (2)	0.0259 (14)	0.071 (2)	−0.0004 (14)	−0.0142 (17)	0.0154 (14)
O6	0.061 (2)	0.0425 (18)	0.074 (2)	−0.0184 (17)	−0.0109 (19)	−0.0271 (17)
O7	0.0337 (18)	0.0453 (18)	0.061 (2)	−0.0087 (15)	−0.0182 (17)	0.0238 (15)
O8	0.0377 (17)	0.0431 (16)	0.0277 (15)	−0.0052 (14)	−0.0104 (13)	−0.0040 (12)
N1	0.096 (4)	0.106 (5)	0.052 (3)	−0.064 (4)	−0.014 (3)	0.014 (3)
N2	0.077 (3)	0.0324 (19)	0.047 (2)	−0.025 (2)	−0.012 (2)	−0.0010 (17)
N3	0.047 (3)	0.0211 (17)	0.087 (3)	−0.0036 (17)	0.006 (2)	−0.0008 (19)
C1	0.045 (3)	0.0304 (19)	0.028 (2)	−0.0130 (18)	−0.0115 (18)	0.0008 (16)
C2	0.041 (3)	0.035 (2)	0.032 (2)	−0.0134 (19)	−0.0137 (18)	0.0021 (17)
C3	0.046 (3)	0.050 (3)	0.032 (2)	−0.025 (2)	−0.011 (2)	0.0033 (19)
C4	0.042 (3)	0.073 (3)	0.042 (3)	−0.027 (3)	−0.012 (2)	−0.005 (2)
C5	0.086 (4)	0.069 (3)	0.033 (3)	−0.055 (3)	−0.010 (3)	−0.001 (2)
C6	0.071 (4)	0.058 (3)	0.052 (3)	−0.032 (3)	−0.029 (3)	0.019 (2)
C7	0.052 (3)	0.047 (3)	0.047 (3)	−0.018 (2)	−0.018 (2)	0.010 (2)
C8	0.040 (2)	0.037 (2)	0.025 (2)	−0.0137 (19)	−0.0115 (18)	0.0023 (16)
C9	0.041 (2)	0.0311 (19)	0.026 (2)	−0.0114 (18)	−0.0125 (17)	−0.0005 (15)
C10	0.041 (3)	0.033 (2)	0.029 (2)	−0.0115 (18)	−0.0075 (18)	−0.0031 (16)
C11	0.045 (3)	0.040 (2)	0.029 (2)	−0.020 (2)	−0.0084 (19)	0.0022 (17)
C12	0.064 (3)	0.0270 (19)	0.027 (2)	−0.018 (2)	−0.016 (2)	0.0047 (16)
C13	0.052 (3)	0.030 (2)	0.036 (2)	−0.006 (2)	−0.006 (2)	−0.0047 (17)
C14	0.043 (3)	0.041 (2)	0.028 (2)	−0.014 (2)	−0.0059 (18)	−0.0004 (17)
C15	0.031 (2)	0.0228 (18)	0.045 (2)	−0.0109 (17)	−0.0009 (18)	−0.0006 (17)
C16	0.029 (2)	0.0219 (17)	0.0278 (19)	−0.0073 (15)	−0.0100 (16)	−0.0011 (14)
C17	0.041 (3)	0.031 (2)	0.038 (2)	−0.0037 (18)	−0.0210 (19)	0.0019 (17)
C18	0.044 (3)	0.036 (2)	0.050 (3)	−0.011 (2)	−0.017 (2)	−0.013 (2)
C19	0.035 (2)	0.0201 (17)	0.050 (3)	−0.0087 (17)	−0.0014 (19)	0.0031 (17)
C20	0.043 (3)	0.039 (2)	0.043 (3)	−0.002 (2)	−0.016 (2)	0.0157 (19)
C21	0.044 (3)	0.040 (2)	0.030 (2)	−0.0086 (19)	−0.0168 (19)	0.0002 (17)
O9	0.067 (3)	0.148 (5)	0.066 (3)	−0.019 (3)	−0.016 (2)	0.010 (3)

### Geometric parameters (Å, º)

**Table:**

Tm1—O5	2.216 (3)	C3—C4	1.388 (6)
Tm1—O6^i^	2.223 (3)	C3—H3	0.9300
Tm1—O7	2.293 (3)	C4—C5	1.405 (7)
Tm1—O2	2.329 (3)	C4—H4	0.9300
Tm1—O3	2.374 (3)	C5—C6	1.376 (6)
Tm1—O8	2.382 (3)	C6—C7	1.355 (7)
Tm1—O4	2.457 (3)	C6—H6	0.9300
Tm1—O1	2.471 (3)	C7—H7	0.9300
Tm1—C1	2.774 (4)	C8—C9	1.488 (5)
Tm1—C8	2.781 (4)	C9—C10	1.387 (6)
O1—C1	1.272 (5)	C9—C14	1.397 (5)
O2—C1	1.270 (5)	C10—C11	1.379 (5)
O3—C8	1.262 (5)	C10—H10	0.9300
O4—C8	1.275 (5)	C11—C12	1.383 (6)
O5—C15	1.253 (5)	C11—H11	0.9300
O6—C15	1.251 (5)	C12—C13	1.397 (6)
O6—Tm1^i^	2.223 (3)	C13—C14	1.377 (6)
O7—H7A	0.817 (10)	C13—H13	0.9300
O7—H7B	0.816 (10)	C14—H14	0.9300
O8—H8A	0.820 (10)	C15—C16	1.487 (5)
O8—H8B	0.819 (10)	C16—C21	1.375 (5)
N1—C5	1.381 (7)	C16—C17	1.383 (5)
N1—H1A	0.847 (10)	C17—C18	1.381 (5)
N1—H1B	0.850 (10)	C17—H17	0.9300
N2—C12	1.399 (5)	C18—C19	1.372 (6)
N2—H2A	0.850 (10)	C18—H18	0.9300
N2—H2B	0.850 (10)	C19—C20	1.378 (6)
N3—C19	1.423 (5)	C20—C21	1.387 (6)
N3—H3A	0.851 (10)	C20—H20	0.9300
N3—H3B	0.842 (10)	C21—H21	0.9300
C1—C2	1.467 (6)	O9—H9A	0.832 (10)
C2—C3	1.379 (6)	O9—H9B	0.835 (10)
C2—C7	1.397 (6)		
			
O5—Tm1—O6^i^	108.53 (13)	O1—C1—C2	121.6 (4)
O5—Tm1—O7	156.90 (13)	O2—C1—Tm1	56.5 (2)
O6^i^—Tm1—O7	84.40 (13)	O1—C1—Tm1	62.9 (2)
O5—Tm1—O2	125.29 (12)	C2—C1—Tm1	171.2 (3)
O6^i^—Tm1—O2	81.97 (13)	C3—C2—C7	117.4 (4)
O7—Tm1—O2	74.42 (11)	C3—C2—C1	122.8 (4)
O5—Tm1—O3	80.05 (12)	C7—C2—C1	119.8 (4)
O6^i^—Tm1—O3	161.28 (12)	C2—C3—C4	121.4 (4)
O7—Tm1—O3	93.61 (13)	C2—C3—H3	119.3
O2—Tm1—O3	79.58 (11)	C4—C3—H3	119.3
O5—Tm1—O8	78.30 (11)	C3—C4—C5	119.4 (5)
O6^i^—Tm1—O8	74.11 (12)	C3—C4—H4	120.3
O7—Tm1—O8	87.42 (11)	C5—C4—H4	120.3
O2—Tm1—O8	151.26 (11)	C6—C5—N1	120.8 (5)
O3—Tm1—O8	124.48 (11)	C6—C5—C4	119.2 (5)
O5—Tm1—O4	80.34 (11)	N1—C5—C4	120.0 (6)
O6^i^—Tm1—O4	142.65 (11)	C7—C6—C5	120.2 (5)
O7—Tm1—O4	78.06 (11)	C7—C6—H6	119.9
O2—Tm1—O4	123.35 (10)	C5—C6—H6	119.9
O3—Tm1—O4	53.82 (10)	C6—C7—C2	122.3 (5)
O8—Tm1—O4	72.41 (10)	C6—C7—H7	118.8
O5—Tm1—O1	75.80 (11)	C2—C7—H7	118.8
O6^i^—Tm1—O1	76.29 (11)	O3—C8—O4	119.1 (4)
O7—Tm1—O1	126.75 (11)	O3—C8—C9	120.4 (4)
O2—Tm1—O1	54.20 (10)	O4—C8—C9	120.4 (4)
O3—Tm1—O1	90.29 (10)	O3—C8—Tm1	58.3 (2)
O8—Tm1—O1	131.47 (10)	O4—C8—Tm1	62.0 (2)
O4—Tm1—O1	139.92 (9)	C9—C8—Tm1	166.2 (3)
O5—Tm1—C1	101.68 (12)	C10—C9—C14	118.4 (4)
O6^i^—Tm1—C1	75.71 (12)	C10—C9—C8	121.6 (4)
O7—Tm1—C1	100.03 (12)	C14—C9—C8	119.8 (4)
O2—Tm1—C1	27.06 (11)	C11—C10—C9	121.1 (4)
O3—Tm1—C1	86.36 (11)	C11—C10—H10	119.4
O8—Tm1—C1	148.00 (11)	C9—C10—H10	119.4
O4—Tm1—C1	139.52 (10)	C10—C11—C12	120.6 (4)
O1—Tm1—C1	27.28 (10)	C10—C11—H11	119.7
O5—Tm1—C8	75.79 (12)	C12—C11—H11	119.7
O6^i^—Tm1—C8	169.50 (13)	C11—C12—C13	118.7 (4)
O7—Tm1—C8	88.48 (13)	C11—C12—N2	120.6 (4)
O2—Tm1—C8	103.56 (11)	C13—C12—N2	120.6 (4)
O3—Tm1—C8	26.88 (11)	C14—C13—C12	120.7 (4)
O8—Tm1—C8	97.92 (11)	C14—C13—H13	119.7
O4—Tm1—C8	27.29 (11)	C12—C13—H13	119.7
O1—Tm1—C8	114.19 (11)	C13—C14—C9	120.5 (4)
C1—Tm1—C8	113.24 (12)	C13—C14—H14	119.8
C1—O1—Tm1	89.8 (3)	C9—C14—H14	119.8
C1—O2—Tm1	96.4 (2)	O6—C15—O5	123.8 (4)
C8—O3—Tm1	94.8 (2)	O6—C15—C16	117.8 (4)
C8—O4—Tm1	90.7 (2)	O5—C15—C16	118.4 (4)
C15—O5—Tm1	145.2 (3)	C21—C16—C17	118.6 (3)
C15—O6—Tm1^i^	157.5 (3)	C21—C16—C15	121.1 (3)
Tm1—O7—H7A	134 (3)	C17—C16—C15	120.3 (3)
Tm1—O7—H7B	118 (3)	C18—C17—C16	120.9 (4)
H7A—O7—H7B	108 (4)	C18—C17—H17	119.6
Tm1—O8—H8A	113 (3)	C16—C17—H17	119.6
Tm1—O8—H8B	139 (3)	C19—C18—C17	120.3 (4)
H8A—O8—H8B	106 (3)	C19—C18—H18	119.8
C5—N1—H1A	126 (4)	C17—C18—H18	119.8
C5—N1—H1B	107 (5)	C18—C19—C20	119.1 (4)
H1A—N1—H1B	120 (4)	C18—C19—N3	121.7 (4)
C12—N2—H2A	114 (4)	C20—C19—N3	119.2 (4)
C12—N2—H2B	116 (3)	C19—C20—C21	120.5 (4)
H2A—N2—H2B	111 (4)	C19—C20—H20	119.8
C19—N3—H3A	109 (4)	C21—C20—H20	119.8
C19—N3—H3B	110 (4)	C16—C21—C20	120.5 (4)
H3A—N3—H3B	112 (4)	C16—C21—H21	119.7
O2—C1—O1	119.0 (4)	C20—C21—H21	119.7
O2—C1—C2	119.4 (4)	H9A—O9—H9B	100 (4)
			
Tm1—O2—C1—O1	−8.3 (4)	C14—C9—C10—C11	0.1 (6)
Tm1—O2—C1—C2	170.8 (3)	C8—C9—C10—C11	175.8 (4)
Tm1—O1—C1—O2	7.8 (4)	C9—C10—C11—C12	−1.2 (6)
Tm1—O1—C1—C2	−171.3 (3)	C10—C11—C12—C13	0.9 (6)
O2—C1—C2—C3	168.9 (4)	C10—C11—C12—N2	−175.6 (4)
O1—C1—C2—C3	−12.0 (6)	C11—C12—C13—C14	0.6 (6)
O2—C1—C2—C7	−8.5 (6)	N2—C12—C13—C14	177.1 (4)
O1—C1—C2—C7	170.6 (4)	C12—C13—C14—C9	−1.8 (6)
C7—C2—C3—C4	1.5 (6)	C10—C9—C14—C13	1.4 (6)
C1—C2—C3—C4	−176.0 (4)	C8—C9—C14—C13	−174.4 (4)
C2—C3—C4—C5	0.1 (7)	Tm1^i^—O6—C15—O5	23.7 (11)
C3—C4—C5—C6	−2.4 (7)	Tm1^i^—O6—C15—C16	−157.9 (6)
C3—C4—C5—N1	178.0 (5)	Tm1—O5—C15—O6	−34.8 (8)
N1—C5—C6—C7	−177.3 (5)	Tm1—O5—C15—C16	146.8 (4)
C4—C5—C6—C7	3.1 (8)	O6—C15—C16—C21	−6.8 (6)
C5—C6—C7—C2	−1.5 (8)	O5—C15—C16—C21	171.7 (4)
C3—C2—C7—C6	−0.8 (7)	O6—C15—C16—C17	175.6 (4)
C1—C2—C7—C6	176.7 (4)	O5—C15—C16—C17	−5.9 (6)
Tm1—O3—C8—O4	−12.7 (4)	C21—C16—C17—C18	−1.5 (7)
Tm1—O3—C8—C9	164.0 (3)	C15—C16—C17—C18	176.2 (4)
Tm1—O4—C8—O3	12.2 (4)	C16—C17—C18—C19	−1.2 (7)
Tm1—O4—C8—C9	−164.5 (3)	C17—C18—C19—C20	4.0 (7)
O3—C8—C9—C10	166.1 (4)	C17—C18—C19—N3	−177.9 (4)
O4—C8—C9—C10	−17.3 (6)	C18—C19—C20—C21	−4.1 (7)
Tm1—C8—C9—C10	−113.5 (12)	N3—C19—C20—C21	177.8 (4)
O3—C8—C9—C14	−18.2 (6)	C17—C16—C21—C20	1.4 (6)
O4—C8—C9—C14	158.4 (4)	C15—C16—C21—C20	−176.2 (4)
Tm1—C8—C9—C14	62.2 (13)	C19—C20—C21—C16	1.4 (7)

Symmetry code: (i) −*x*+1, −*y*, −*z*.

### Hydrogen-bond geometry (Å, º)

*Cg*3 is the centroid of the C16–C21 ring.

**Table:**

*D*—H···*A*	*D*—H	H···*A*	*D*···*A*	*D*—H···*A*
O7—H7*A*···N3^ii^	0.82 (1)	1.95 (2)	2.753 (6)	169 (5)
O7—H7*B*···N2^iii^	0.82 (1)	2.18 (2)	2.940 (5)	154 (5)
O8—H8*A*···O1^i^	0.82 (1)	1.98 (1)	2.791 (4)	172 (4)
O8—H8*B*···O4^iv^	0.82 (1)	1.96 (1)	2.780 (4)	174 (4)
N1—H1*B*···O9^v^	0.85 (1)	2.06 (3)	2.870 (8)	158 (7)
N2—H2*B*···O9^vi^	0.85 (1)	2.24 (2)	3.051 (7)	161 (5)
N3—H3*B*···O5^vii^	0.84 (1)	2.46 (3)	3.173 (6)	144 (5)
N3—H3*B*···O8^vii^	0.84 (1)	2.56 (5)	3.092 (5)	122 (4)
O9—H9*A*···O3	0.83 (1)	2.00 (1)	2.828 (6)	172 (7)
O9—H9*B*···O2^viii^	0.84 (1)	2.34 (6)	2.849 (6)	119 (5)
C11—H11···*Cg*3^ix^	0.93	2.68	3.538 (5)	155

Symmetry codes: (i) −*x*+1, −*y*, −*z*; (ii) *x*+1, *y*−1, *z*; (iii) *x*, *y*−1, *z*; (iv) −*x*+2, −*y*, −*z*; (v) −*x*, −*y*, −*z*+1; (vi) −*x*+1, −*y*+1, −*z*+1; (vii) −*x*+1, −*y*+1, −*z*; (viii) −*x*+1, −*y*, −*z*+1; (ix) *x*+1, *y*, *z*.
